# ﻿*Rosadavurica* var. ﻿*rubro-stipulata* (Rosaceae), the correct name for ﻿*R.davurica* var. ﻿*﻿alpestris*

**DOI:** 10.3897/phytokeys.229.105786

**Published:** 2023-07-07

**Authors:** Young-Soo Kim, Dong Chan Son

**Affiliations:** 1 Division of Forest Biodiversity and Herbarium, Korea National Arboretum, Pocheon 11186, Republic of Korea Korea National Arboretum Pocheon Republic of Korea

**Keywords:** autonym, nomenclature, priority, Shenzhen Code

## Abstract

The name Rosadavuricavar.alpestris (Nakai) Kitag. was published in 1979 as a new combination based on R.rubro-stipullatavar.alpestris Nakai. It is generally accepted as a deciduous shrub occurring in Russia, Manchuria, Japan, and the northern part of the Korean Peninsula and is distinguished by the presence of eglandular leaves. Rosarubro-stipullatavar.alpestris was originally described as a new variety with a leaf size relatively smaller than that of R.rubro-stipullatavar.rubro-stipullata . However, the observation of various specimens showed the leaf size of var.alpestris to be of minor importance, and it was included in var.rubro-stipullata as a synonym. Due to the priority of autonyms, a new combination is required to replace R.davuricavar.alpestris. Additionally, it should be noted that the epithet “*rubro-stipullata*” is derived from the Latin word “*stipula*” rather than “*stipulla*.” Therefore, for this variety, we propose a new combination, R.davuricavar.rubro-stipulata (Nakai) D. C. Son & Y. S. Kim, **comb. nov. & stat. nov.**

Rosadavuricavar.alpestris (Nakai) Kitag. is a deciduous shrub distributed through Russia, Manchuria, Japan, and the northern part of the Korean Peninsula. *Rosadavurica* Pall. is remarkable because of the variable shape of its leaflets, from narrowly to broadly elliptic, and the absence or presence of glands on their lower surface. The varietal name is commonly applied to plants of *R.davurica* with eglandular leaflets (Kitagawa 1979; Lee 2003; Ohba 2001). Further morphological observations showed that var. alpestris is readily distinguished from var. davurica by the presence of eglandular rachis and petiole, abaxial surface of calyx lobe sparsely glandular or eglandular, and flower 2~3 cm in diam. (Fig. 1; Table 1).

**Figure 1.**
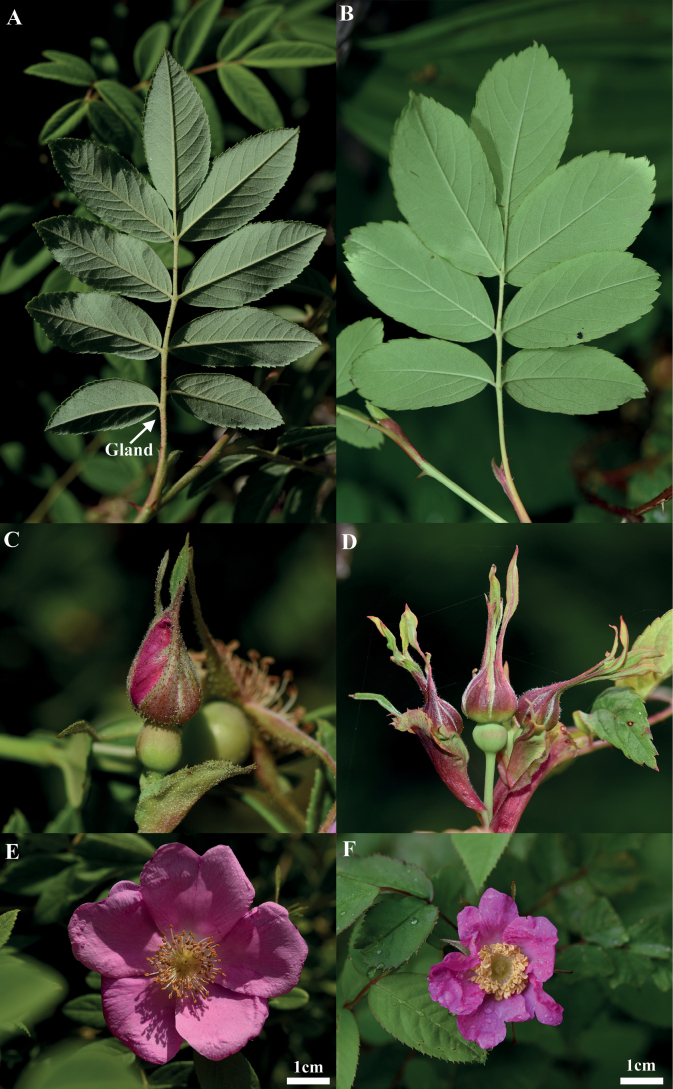
Morphological characters distinguishing *R.davurica* (**A, C, E**) and R.davuricavar.alpestris (**B, D, F**) **A, B** leaves **C, D** calyx lobe **E, F** flower. Photo Credits: Dong-Hyuk Lee.

**Table 1.** Morphological differences among *Rosadavurica* and R.davuricavar.alpestris.

	Characters	*R.davurica*	R.davuricavar.alpestris
Leaflet	Presence of gland on abaxial surface	Glandular	Eglandulose
Rachis	Presence of gland on surface	Glandular	Eglandulose
Petiole	Presence of gland on surface	Glandular	Eglandulose
Calyx lobe	Density of hair on abaxial surface	Densely glandular	Sparsely glandular or eglandulose
Flower	Diameter (cm)	4~5	2~3

Rosadavuricavar.alpestris (Nakai) Kitag. was published in 1979, as a new combination based on R.rubro-stipullatavar.alpestris Nakai. Rosarubro-stipullatavar.alpestris Nakai was originally described as a new variety with a leaf size relatively smaller than that of R.rubro-stipullatavar.rubro-stipullata (Nakai 1916). However, based on several specimens, including type specimens of var. alpestris and var. rubro-stipullata, we observed that although the leaf size of Rosaalpestris was smaller than that of var. rubro-stipullata, this character does not correlate with any other morphological trait or geographical feature, and it is not taxonomically worthy of being recognized as a variety. Therefore, it is reasonable to regard R.rubro-stipullatavar.alpestris as a synonym of R.rubro-stipullatavar.rubro-stipullata. In practice, R.rubro-stipullatavar.rubro-stipullata has been treated as a synonym of R.davuricavar.alpestris in the literature (Kitagawa 1979; Ohba 2001; Chang et al. 2014; Korea National Arboretum 2020; POWO 2023; WFO 2023).

**Figure 2.**
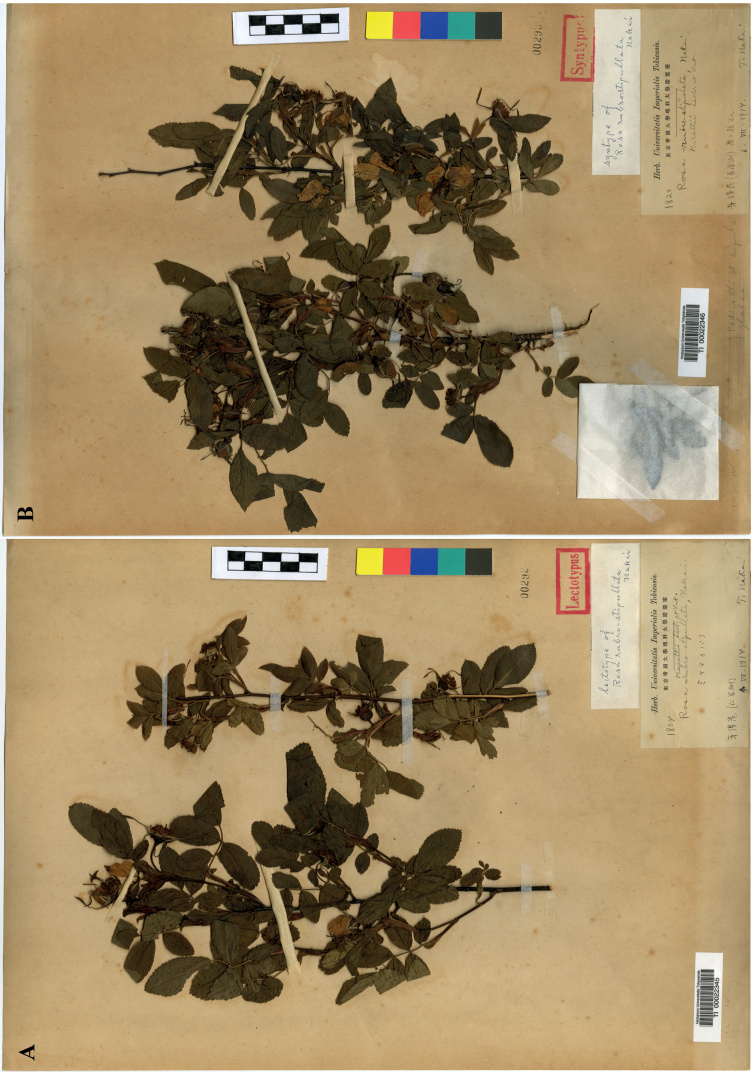
Type specimens of Rosarubro-stipulatavar.rubro-stipulata**A** lectotype (TI00022345) **B** syntype (TI00022346).

According to the rules of the ICN (Turland et al. 2018), “*An autonym is treated as having priority over the name* (*s*) *of the same date and rank that upon their valid publication established the autonym*,” and a new combination is required to replace R.davuricavar.alpestris because of the priority of the autonym (see ICN Article 11.6 Ex. 28). Meanwhile, the epithet “*rubro-stipullata*” is derived from the Latin word “*stipula*” rather than “*stipulla*”, hence it should be corrected to “*rubro-stipulata*” (see ICN Article 60.1). Therefore, for this variety, we propose a new combination, Rosadavuricavar.rubro-stipulata (Nakai) D. C. Son & Y. S. Kim.

**Figure 3.**
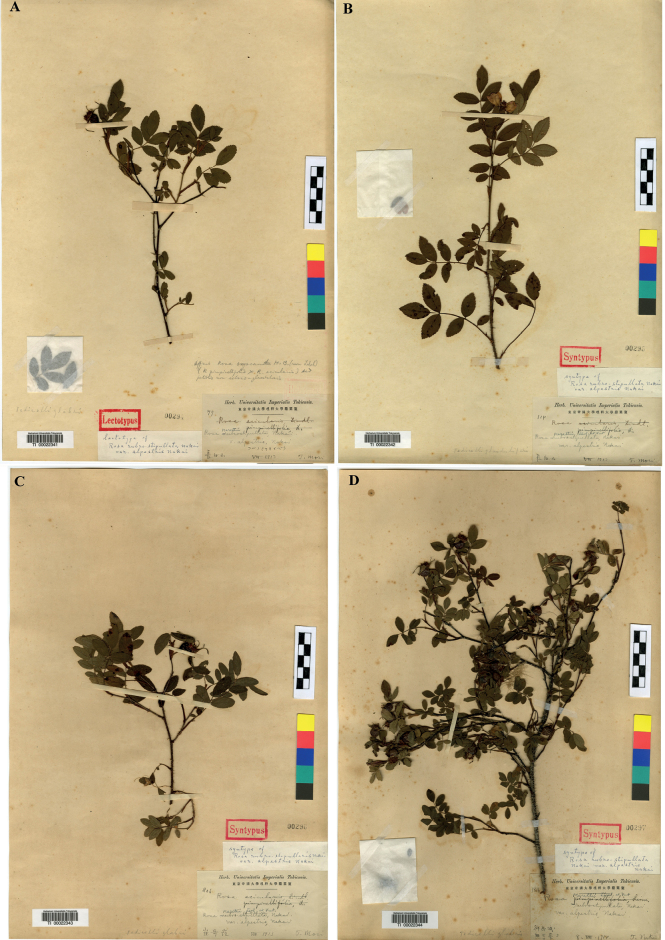
Type specimens of Rosarubro-stipulatavar.alpestris**A** lectotype (TI00022341) **B–D** syntype (TI00022342, TI00022343, TI00022344).

## ﻿Taxonomic treatment

### ﻿ Rosa davurica var. rubro-stipulata

Taxon classificationPlantaeRosalesRosaceae

(Nakai) D. C. Son & Y. S. Kim, comb. nov. & stat. nov.

3E1B6CEE-5447-5E71-9B6B-9476FF107B98

urn:lsid:ipni.org:names:77322794-1

Rosa rubro-stipulata Nakai, Bot. Mag. (Tokyo) 30: 242 (1916). Basionym. **Type.** Korea. Chagang-do: 牙得嶺 (江界側) [Adeuk-ryeong (Ganggye)], July 5, 1914, T. Nakai 1824 (lectotype, designated by Momiyama and Ohba (1988: 10): TI00022345, photo!); KOREA Hamgyongnam-do: 牙得嶺 (長津側) [Adeuk-ryeong (Chang-jyu)], July 6, 1914, T. Nakai 1820 (syntype: TI00022346, photo!). Fig. 2.
= Rosarubro-stipulatavar.alpestris Nakai, Bot. Mag. (Tokyo) 30: 242 (1916); Rosamarretiivar.alpestris (Nakai) Uyeki, Woody Pl. Distr. Chosen: 51 (1940); Rosadavuricavar.alpestris (Nakai) Kitag., Neolin. Fl. Manshur. 382 (1979). **Type.** Korea. Hamgyeongbuk-do: 長白山 (Baekdusan), August 1913, *T. Mori 77* (lectotype, designated by Momiyama and Ohba (1988: 11): TI00022341, photo!); KOREA. Hamgyeongbuk-do: 長白山 (Baekdusan), August 1913, *T. Mori 114* (syntype: TI00022342, photo!); KOREA. Ryanggang-do: 崔哥嶺 (Choiga-ryeong), August 1913, *T. Mori 206* (syntype: TI00022343, photo!); KOREA. Ryanggang-do: 神武城 – 無頭峯 (Shinmusung – Mudubong), August 8, 1914, *T. Nakai 1816* (syntype: TI00022344, photo!). Fig. 3.

## Supplementary Material

XML Treatment for Rosa davurica var. rubro-stipulata

## ﻿Additional information

### Author ORCIDs

Young-Soo Kim https://orcid.org/0000-0002-8524-2441

Dong Chan Son https://orcid.org/0000-0002-6773-0580

### Data availability

All of the data that support the findings of this study are available in the main text.
